# Statins in anthracycline-induced cardiotoxicity: Rac and Rho, and the heartbreakers

**DOI:** 10.1038/cddis.2016.418

**Published:** 2017-01-19

**Authors:** Christian Henninger, Gerhard Fritz

**Affiliations:** 1 Department of Toxicology, Medical Faculty, Heinrich Heine University Düsseldorf, Moorenstrasse 5, D-40225 Düsseldorf, Germany

## Abstract

Cancer patients receiving anthracycline-based chemotherapy are at risk to develop life-threatening chronic cardiotoxicity with the pathophysiological mechanism of action not fully understood. Besides the most common hypothesis that anthracycline-induced congestive heart failure (CHF) is mainly caused by generation of reactive oxygen species, recent data point to a critical role of topoisomerase II beta (TOP2B), which is a primary target of anthracycline poisoning, in the pathophysiology of CHF. As the use of the only clinically approved cardioprotectant dexrazoxane has been limited by the FDA in 2011, there is an urgent need for alternative cardioprotective measures. Statins are anti-inflammatory and anti-oxidative drugs that are clinically well established for the prevention of cardiovascular diseases. They exhibit pleiotropic beneficial properties beyond cholesterol-lowering effects that most likely rest on the indirect inhibition of small Ras homologous (Rho) GTPases. The Rho GTPase Rac1 has been shown to be a major factor in the regulation of the pro-oxidative NADPH oxidase as well as in the regulation of type II topoisomerase. Both are discussed to play an important role in the pathophysiology of anthracycline-induced CHF. Therefore, off-label use of statins or novel Rac1 inhibitors might represent a promising pharmacological approach to gain control over chronic cardiotoxicity by interfering with key mechanisms of anthracycline-induced cardiomyocyte cell death.

## Facts

- Anthracycline-induced cardiotoxicity is an unresolved major problem in cancer therapy.
- Rho GTPases have nuclear functions that might influence the doxorubicin-induced DNA damage response.
- Rho GTPases interfere with two of the supposed main mechanisms of anthracycline-induced cardiotoxicity: generation of reactive oxygen species and topoisomerase II poisoning.
- A preventive treatment with statins or specific inhibitors of Rho GTPases are promising pharmaceutical approaches to alleviate anthracycline-induced cardiotoxicity.

## Open questions

- Does topoisomerase II-mediated mtDNA damage play a role in anthracycline-induced cardiotoxicity?
- How do Rho GTPases regulate topoisomerase II activity?
- Are nuclear functions of Rho GTPases involved in the anthracycline-induced DNA damage response?
- What is more relevant for chronic cardiotoxicity: the generation of reactive oxygen species or topoisomerase II beta poisoning?
- The cardioprotective effects of statins in anthracycline-based chemotherapy needs verification in randomized prospective studies.

Anthracyclines are potent chemotherapeutics, which are used for the treatment of a broad spectrum of malignancies.^1^ The supposed antineoplastic mechanism is the induction of DNA damage, predominantly in the S- and G2-phase of proliferating cells.^2^ Anthracyclines such as epirubicin or doxorubicin inhibit type II topoisomerases, thereby causing DNA double-strand breaks (DSBs),^3^ which represent a strong apoptotic stimulus if left unrepaired.^4, 5^ In addition, anthracyclines intercalate into DNA, form bulky DNA adducts and DNA crosslinks, which interfere with DNA replication and transcription. They can damage DNA directly due to the generation of reactive oxygen species (ROS), leading to oxidized nucleotides, base mismatches, point mutations and DNA single-strand breaks. The production of ROS also causes a DNA damage-independent stimulation of cytotoxic mechanisms, resulting from oxidative protein modifications, in particular, lipid peroxidation.^6, 7^ Last, anthracyclines interfere with DNA helicase activity and DNA strand separation.^8^

Unfortunately, the geno- and cytotoxic effects evoked by anthracyclines are not limited to tumour cells. Adverse effects of anthracycline-based chemotherapy on normal tissue can be severe and dose limiting.^9^ Patients are at considerable risk to develop acute and chronic cardiotoxicity with the mechanism(s) involved under debate. Acute cardiotoxicity during therapy is rare, not dose-related and often associated with pre-existing cardiac diseases.^10, 11^ More common and by far more serious is chronic cardiotoxicity, which can occur weeks or even years after treatment. In >50% of patients who survived childhood leukaemia echocardiographic abnormalities are detectable after anthracycline-based therapeutic regimen.^12^ Chronic cardiotoxicity usually manifests during the first year after the end of anthracycline treatment but can also occur decades later.^13, 14, 15, 16, 17, 18, 19^

Breast cancer patients treated with the anthracycline-derivative doxorubicin showed decreased left ventricular ejection fraction (LVEF) when the cumulative doxorubicin dose exceeded 350 mg/m^2^ (refs 20, 21). In a retrospective study comprising 4000 patients, 88 developed congestive heart failure (CHF) after treatment. The incidence ranged from 0.1 to 7.0% depending on the cumulative dose (<400–550 mg/m^2^). In patients receiving ≥700 mg/m^2^ the incidence was 18%.^22^ In consequence of these data, reduction of the maximum cumulative dose to 550 mg/m^2^ was recommended, which unfortunately is accompanied by reduced anti-tumour efficiency. Notably, even when adhering to the suggested maximum doxorubicin dose, ~26% of patients are at risk to develop CHF.^9^ A cohort study of adult survivors of childhood leukaemia found that these patients have a twofold higher risk of developing CHF when having received a cumulative dose of <250 mg/m^2^ and a fivefold higher risk when >250 mg/m^2^ were applied (compared to patients who received a non-anthracycline-based therapy).^23^

## Mechanisms of anthracycline-induced cardiotoxicity

A hallmark of anthracycline-induced chronic cardiotoxicity is the reduction of left ventricular wall thickness due to the loss of cardiomyocytes, resulting in restricted LVEF.^24^ Anthracycline-induced cardiomyocyte cell death is likely mediated through caspase-3-related apoptotic pathways activated by p53 and/or TNF-signalling.^25^ The trigger stimuli ultimately causing cardiomyocyte cell death are uncertain and controversially discussed. Suggested mechanisms for the development of cardiomyopathy include accumulation of toxic metabolites (e.g., doxorubinicol), autophagy, production of peroxynitrite and ROS, TOP2B inhibition, and disruption of mitochondrial homeostasis/integrity.^8, 26^ A detailed discussion of each of these putative factors is beyond the scope of this review. Only the most common original hypothesis and the latest most topical hypothesis, for which new evidence was found recently, are highlighted here.

### The ‘ROS and iron' hypothesis

**Figure 1:**
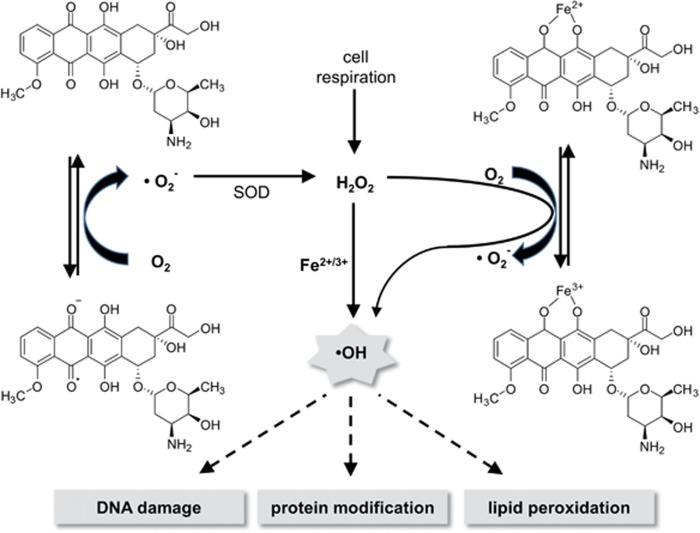
Model of doxorubicin-mediated generation of reactive oxygen species (ROS) by redox cycling and Fenton‘s reaction. Doxorubicin produces ROS by redox cycling at its semiquinone structure. In the Fenton‘s reaction, iron catalyses the generation of OH radicals (•OH) from H_2_O_2_. SOD, superoxide dismutase; adapted from Simunek *et al.*^6^ and Stêrba *et al.*^7^

Cardiomyocytes are described to be particular sensitive to ROS. It is believed that their anti-oxidative defence systems are saturated by endogenous oxidative metabolism.^27^ Additional ROS exposure could exhaust this system faster as compared to other tissues. Anthracyclines could cause such additional ROS generation because they are reductively activated to a semiquinone radical, which undergoes redox cycling, thereby producing superoxide (O_2_^−^) and hydrogen peroxide (H_2_O_2_). In the presence of iron, anthracyclines can form Fe^3+^–anthracycline complexes, which further catalyse the conversion of H_2_O_2_ to various ROS species, including cytotoxic hydroxyl radicals (OH^−^; Fenton's reaction). Redox cycling from a single anthracycline molecule could lead to the accumulation of ROS^7^ (Figure 1). This so-called ‘ROS and iron' hypothesis is highly popular to explain the cardiotoxicity of anthracyclines, but the postulated relevance for chronic cardiotoxicity lacks convincing experimental evidence. Besides, it is under debate whether ROS formation plays a predominant pathophysiological role when clinically relevant (low) doses of anthracyclines are used. Importantly, most of the *in vitro* and *in vivo* studies demonstrating ROS formation after doxorubicin treatment used supra-clinical doses that are magnitudes higher than the concentrations detected in the serum of patients during chemotherapy.^6, 8, 28^ *In vitro* studies with rat cardiomyocytes showed that anthracycline-induced ROS formation was very low or even below detection limit at clinically relevant doses (≤2 *μ*M doxorubicin).^29, 30^

Transgenic mice that overexpress human manganese-dependent superoxide dismutase (MnSOD) in the heart are protected from doxorubicin-induced acute cardiotoxicity, pointing to a critical role of ROS as inducer of acute cardiac damage and MnSOD as major detoxification mechanism.^31^ However, the doses of 10–25 mg/kg doxorubicin used in this study are equivalent to the 1–2.5-fold LD_50_ of mice.^32^ There are plenty of studies addressing acute toxic anthracycline effects after high dosage and short follow-up time that claim the relevance of ROS, anti-oxidative drugs or ROS-detoxifying enzymes.^6^ Yet, none of them proves a pivotal role of ROS in the pathophysiology of chronic cardiotoxicity observed under clinical relevant settings of anthracycline-based anti-cancer therapy. Furthermore, it is hard to distinguish whether ROS generation is the primary mode of cardiotoxic action of anthracyclines or a secondary phenomenon resulting from cardiotoxicity. For instance, enhanced ROS levels can also result from mitochondrial dysfunction or endoplasmatic reticulum stress caused by accumulation of misfolded proteins.^33^ Another point to be considered is that anthracyclines exhibit a strong auto-fluorescence.^34, 35^ As common ROS detection methods are fluorescence-based, this can lead to the misinterpretation of results, in particular when high doses (>2 *μ*M) of anthracyclines are used. Down to the present day, it is unclear whether or not generation of ROS occurs in relevant amounts under standard anthracycline treatment regimens, undermining its role as main trigger of chronic cardiotoxicity.

**Figure 2:**
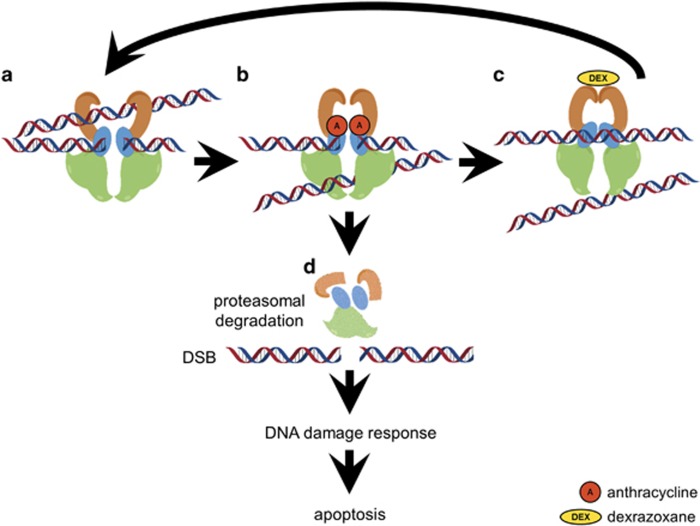
Inhibition of type II topoisomerases by anthracyclines and dexrazoxane. To resolve tensions in supercoiled DNA, topoisomerase II induces a DNA double-strand break (DSB) to allow passage of another DNA strand, which is delivered from the open clamp (orange) (**a**). The DNA strand passes downwards through the DSB of the first strand and subsequently the open strand is resealed again (**c**). If anthracyclines bind at the region of interplay between the opened DNA and the topoisomerase II (**b**), the resealing of the DSB is disabled and a so-called cleavable complex consisting of anthracycline, DNA and topoisomerase persists until it is proteasomally degraded (**d**). Consequently, the opened DNA is left over containing a highly cytotoxic DSB that can trigger a pro-apoptotic DNA damage response. Binding of dexrazoxane to the ATPase domain of the topoisomerase keeps its clamp in a closed state (**c**), which prevents anthracycline binding as well as entrance into another enzymatic cycle (transition from the state illustrated under **c** to the state illustrated under **a**). A, anthracycline; DEX, dexrazoxane; DSB, DNA double-strand break; adapted from Lyu *et al.*^54^ and Vejpongsa and Yeh^39^

Application of antioxidants and iron-chelating agents led to inconclusive results in animal experiments. Importantly, no protection from anthracycline-induced cardiotoxicity was found in patients treated with *N*-acetyl cysteine or combinations of antioxidants.^6^ By contrast, the iron-chelating drug dexrazoxane (ICRF-187), which is structurally related to EDTA, effectively alleviated anthracycline-induced cardiotoxicity in numerous animal and human studies. Yet, the precise cytoprotective mechanism of dexrazoxane remains unclear and studies with other iron chelators failed to show cardioprotection.^6^ Apart from iron chelation, which inhibits the production of hydroxyl radicals resulting from Fenton's reaction, dexrazoxane also inhibits type II topoisomerases^36, 37, 38, 39, 40, 41^ by a catalytic mechanisms that is different from anthracycline poisoning and does not comprise cleavable complex formation^39, 40, 42^ (Figure 2). In light of this, it is feasible that dexrazoxane does not solely bind free iron, but may also lower the amount of the accessible main substrate (i.e., topoisomerase II) for anthracycline poisoning.^39, 40^ This could result in reduced cardiotoxicity but may also impair the anti-tumour efficiency of anthracyclines. Early clinical studies with dexrazoxane dispelled those concerns, as tumour protection was not observed.^43, 44^ However, two recent studies claimed that dexrazoxane bears the risk to affect anthracycline's anti-tumour efficiency and to increase the incidence of secondary tumours.^45, 46^

### Type II topoisomerase poisoning

Proliferating cells depend on topoisomerase II alpha (TOP2A) for chromosome condensation and segregation.^47, 48^ The beta form (TOP2B) predominantly contributes to transcription.^49^ Knockout of the alpha form revealed that the beta form may substitute for its function in chromosome condensation, but cannot complement for chromosome segregation.^50^ Proliferating cells show an enhanced expression of the alpha form in S- and G2-phase, whereas its amount is low in G0/G1 phase cells.^51^ The beta isoform is constitutively expressed independent of the cell cycle.^52^ Type II topoisomerases cut DNA double-strands to allow strand passage for unwinding and unknotting of supercoiled DNA (Figure 2). Typically, these cuts are resealed right after strand passage.^39, 40, 53^ Upon poisoning of topoisomerase by anthracyclines, resealing of the transient DSB becomes impossible. The anthracycline stabilizes the temporary cleavage complex consisting of DNA and topoisomerase to a permanently stalled complex. Presumably, this complex is proteasomally degraded and DNA DSBs remain.^54^ Like stated above, accumulation of non-repaired DSBs represents a strong apoptotic stimulus^4, 5, 55^ that can trigger cardiomyocyte death.

Cardiomyocytes lack the expression of TOP2A but harbour relative high levels of TOP2B as compared to other tissues.^56^ This might render them particular vulnerable to topoisomerase II poisons such as anthracyclines. In line with this, Lyu *et al.*^54^ showed that knockout of TOP2B protects mouse embryonal fibroblasts from anthracycline-induced cytotoxicity. In addition, the expression level of TOP2B correlates with anthracycline-induced apoptosis in peripheral blood cells.^57^ Most importantly, mice with a cardiac-specific *top2b* knockout exhibit less cardiotoxicity following anthracyline treatment.^58^ The *top2b* knockout mice revealed lower levels of DSBs, no decline in LVEF after doxorubicin treatment and showed reduced mitochondrial dysfunction. These results support the hypothesis that the cardioprotective effects seen with dexrazoxane are at least partially dependent on its interference with topoisomerase II.

Another interesting aspect related to TOP2 poisoning by anthracyclines is the fact that both TOP2A and TOP2B were found in the mitochondria recently.^59, 60^ As the beta form is predominantly expressed in the heart, it is likely the relevant form in cardiac mitochondria too.^60^ Anthracyclines can induce mitochondrial damage due to uncoupling of the electron transport chain, disruption of mitochondrial membrane potential and production of ROS, especially in combination with the mitochondrial iron metabolism.^61^ As anthracyclines induce DSBs in genomic DNA via topoisomerase II poisoning, it is feasible that a similar genotoxic mechanism also occurs in mitochondrial DNA (mtDNA) and contributes to anthracycline-induced cytotoxicity. As CHF occurs at late times after anthracycline treatment, a long-lasting mitochondrial dysfunction (and concomitant sustained increase in the steady-state levels of ROS) may add to the pathophysiology of chronic cardiotoxicity. Indeed, studies in mice and rabbits demonstrated that chronic anthracycline-induced heart failure correlates with imbalances in mitochondrial mass and reduced expression of genes regulating mitochondrial homeostasis.^62, 63^ However, whether TOP2-dependent DSBs in mtDNA or TOP2-independent oxidative mtDNA base lesions cause depletion of mtDNA content is hard to discriminate.

Recently, Jean *et al.*^64^ showed that mice treated with a chemically modified doxorubicin derivative, which exclusively enters mitochondria but not the nucleus, lack cardiotoxicity, arguing against a dominant contribution of mtDNA damage in the development of cardiomyopathy following anthracycline treatment. The authors suggest that altered nuclear expression of genes encoding mitochondrial biogenesis factors such as NRF1 and TFAM, is more relevant for the onset of cardiotoxicity than direct mitochondrial damage. Recently, Khiati *et al.*^65^ showed that mice lacking mitochondrial type I topoisomerase (mtTOP1), are hypersensitive to anthracyclines. As mtTOP1 is required to maintain efficient mtDNA production after mitotoxic insults, this finding supports the idea of anthracycline-induced mtDNA damage being of relevance for the pathophysiology of chronic cardiotoxicity. Regardless of whether or not TOP2 poisoning of mitochondrial or genomic DNA plays a key role in anthracycline-evoked cardiotoxicity, TOP2B seems to be a promising pharmacological target for its prevention. Pharmacological inhibition of TOP2B during the time period anthracyclines are applied might protect non-proliferating cardiac cells, whereas TOP2A would still be poisoned in proliferating tumour cells.

## Current treatment and prevention strategies

As the use of dexrazoxane for the prevention of anthracycline-induced CHF was restricted by the FDA in 2011, alternative cardio-preventive measures, especially for cardioprophylaxis in children, are urgently needed. Cardioprotective preventive measures used today are limitation of the cumulative anthracycline dose and application of ‘less cardiotoxic' derivatives or liposomal encapsuled forms. The therapy of anthracycline-induced CHF matches that of anthracycline-independent CHF and includes ACE inhibitors, beta-blockers, diuretics, aldosterone blockers and cardiac glycosides.^66^ The therapeutic benefit closely depends on the improvement of left ventricular function.^67, 68^ The ACE inhibitor enalapril and the beta-blocker carvedilol are the most effective drugs in achieving normalization of anthracycline-caused decrease in LVEF.^69^ Due to these promising therapeutic results, a preventive study was initiated. In the OVERCOME trail, 42% of the patients showed a preservation of LVEF by prophylactic enalapril and carvedilol treatment, and 10% of patients responded partially.^70^ However, these cardioprotective effects are less marked than in the case of dexrazoxane-based prevention.

## Statins in the prevention of cardiovascular disease

Statins (HMG-CoA reductase inhibitors) are first choice for the treatment of hypercholesterolemia since 1987.^71^ Elevated blood cholesterol levels are a major risk factor for atherosclerosis.^72^ Following cholesterol accumulation on blood vessel walls (so-called plaques) and macrophage infiltration, the constriction of blood vessels is promoted.^73^ Statins competitively inhibit the conversion of HMG-CoA to mevalonate, a precursor for cholesterol synthesis,^74^ thereby eventually reducing the cell's intrinsic cholesterol synthesis and favouring the uptake of serum LDL cholesterol.^75^ As the mevalonate pathway is essential for the synthesis of numerous isoprenoids, statins have multiple so-called pleiotropic effects that are independent of cholesterol biosynthesis.^76, 77^

### Non-lipid-lowering effects of statins

**Figure 3:**
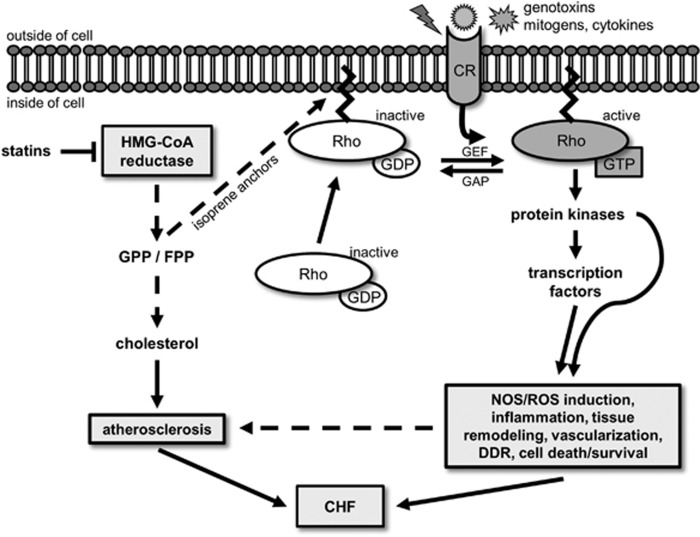
Indirect inhibition of Rho GTPase signaling by HMG-CoA reductase inhibitors (statins). Non-cholesterol-related beneficial effects of statins mainly rest on the indirect inhibition of Rho GTPases. The statin-mediated depletion of the intracellular pool of isoprene precursors causes reduced anchorage of Rho proteins on the inner cell membrane, which impairs their activation by GEFs on this particular location. Depicted are selected cellular functions regulated by Rho GTPases that might be relevant for the heart. CHF, congestive heart failure; CR, cellular receptor; DDR, DNA damage response; FPP, farnesyl pyrophosphate; GAP, GTPase activating protein; GDP, guanosine diphosphate; GEF, guanine nucleotide exchange factor; GGP, geranylgeranyl pyrophosphate; GTP, guanosine triphosphate; NOS, nitric oxide synthase; Rho, Ras homologous proteins; ROS, reactive oxygen species; adapted from Fritz *et al.*^155^

Statins prevent from cholesterol-related plaque generation, reduce already formed plaques, prevent their breakage and attenuate thrombocyte-mediated vessel blockage.^78, 79^ In addition, they exhibit anti-inflammatory properties and reduce deleterious heart tissue remodelling.^80, 81^ These beneficial effects cannot be explained solely by changes in LDL/HDL ratios. Rather, they were mostly traced back to the inhibition of small Ras homologous (Rho) GTPases.^75^ Rho GTPases are molecular switches and signal transducers on the inner cell membrane that transmit extracellular stimuli to mitogen-activated protein kinases and transcription factors.^82, 83^ Inhibition of the HMG-CoA reductase causes depletion of the cellular pool of isoprene precursors, which are also essential for C-terminal prenylation and subsequent membrane localization of Rho proteins^84, 85, 86, 87^ (Figure 3). Rho GTPases are famous as key regulators of the actin-cytoskeleton, but exhibit manifold functions beyond that.^88, 89, 90^ For instance, RhoA is involved in the regulation of endothelial cell migration, blood vessel tension and thrombocyte aggregation.^91, 92, 93^ The RhoA downstream effector Rho-associated coiled-coil forming protein kinase (ROCK) regulates the activity of the endothelial nitric oxide synthase (eNOS).^94^ RhoA/ROCK inhibition or treatment with statins stabilizes eNOS mRNA as well as AKT-mediated activation of eNOS.^95, 96^ The release of nitric oxide reduces endothelial tension, vascular inflammation, thrombocyte aggregation and thus works against atherosclerosis.^92, 95, 97^ Rac1 is part of the NADPH oxidase complex, which initiates pro-inflammatory processes.^98, 99^ In endothelial cells, Rac1 is a major regulator of migration, adhesion and controls vascularization.^100^ Taken together, attenuation of Rho GTPase signalling seems to contribute to the anti-atherosclerotic properties of statins and might also be of relevance beyond the maintenance of cardiovascular health.

A quite novel aspect is the possible role of Rho GTPases in the regulation of the DNA damage response (DDR).^101, 102, 103, 104^ The DDR is a complex and fine-tuned network of signalling cascades that are involved in recognition of DNA damage, DNA repair, cell cycle progression, cell death and survival.^4, 5, 105^ Surprisingly, the RhoA-specific guanine nucleotide exchange factor (GEF) Net1 was found in the nucleus,^103^ implicating nuclear functions of RhoA. GEFs are a group of proteins that mediate GTP-binding and thereby the activation of Rho GTPases^106, 107^ (Figure 3). For Rac1, cell cycle-dependent nuclear translocation was shown and associated with mitosis.^108^ Moreover, it was shown that prevention of Rac1 prenylation by statins promotes Rac1 translocation to the nucleus,^108^ pointing to statin-mediated loss of cytosolic function of Rac1 going along with a gain of nuclear function. Recent data suggest that nuclear Rac1 might be involved in the regulation of topoisomerase II activity and the DDR.^102, 104, 109^ The latter is supported by the finding that oxidized DNA bases can act as Rac1 GEFs.^110^ Taken together, inhibition of Rho proteins such as RhoA or Rac1 by statins also modulates mechanisms of the DDR independent of cholesterol metabolism. The pleiotropic, cardioprotective characteristics of statins and their favourable tolerability make them promising candidates for the prevention and/or treatment of anthracycline-induced cardiotoxicity.

### Statins and anthracycline-induced cardiotoxicity

In 2000 Felezko *et al.* tested the anti-tumour potential of lovastatin if combined with doxorubicin in mouse xenograft models.^111^ The statin improved the anti-tumour efficiency of the anthracycline. In addition, co-treated mice showed less troponin T release, pointing to cardioprotective effects of the statin. This finding implies that statins foster a win–winsituation in anthracycline-based chemotherapy: sensitizing tumour cells while protecting the heart. Indeed, statins are described to sensitize different tumour entities against various chemotherapeutics in rodent models^112, 113, 114, 115, 116, 117^ and are useful in chemoprevention of colorectal cancer.^118^

In rats, rosuvastatin was more potent to protect from doxorubicin-induced delayed cardiotoxicity than carvedilol.^119^ The beneficial effects seemed to be independent of the anti-oxidative properties of the statin. By contrast, Riad *et al.* suggested both anti-oxidative and anti-inflammatory effects of statins to contribute to cardioprotection. The statin enhanced SOD2 levels, reduced caspase-3-mediated apoptosis and mitigated cardiac inflammation following doxorubicin treatment.^120^ However, their experimental set-up was designed for the molecular analysis of acute toxic effects shortly after high doses of doxorubicin and statin. Transferring these results to the chronic situation is complicated. Noteworthy, certain statins show anti-oxidative capacity (e.g., atorvastatin, simvastatin or fluvastatin)^121^ and activate anti-oxidative Keap1/Nrf2 signalling.^122^ Nrf2 is responsible for the expression of genes coding for anti-oxidative factors (i.e., SOD, GST or GSH).^123^ Like stated above, the small Rho GTPase Rac1 is a key regulator of the NADPH oxidase complex. This complex is a major factor in the defence against endo-parasites but is also involved in the regulation of endothelial functions and vascular tone.^124, 125^ Immune cells such as macrophages use this complex to produce superoxide anions that are released to the intercellular space. The resulting oxidative stress can be a driver of chronic inflammation with deregulated macrophage activity.^126, 127^ Mice with impaired Nox2 NADPH oxidase complex are less sensitive to anthracycline-induced cardiotoxicity, pointing to a crucial role of this Rac1-regulated enzyme in the pathology of cardiotoxicity evoked by anthracyclines.^128^ Moreover, a Rac1 knockout in cardiomyocytes of mice prevents angiotensin II-induced cardiac hypertrophy, which also involves NADPH oxidase activation.^129^ It is tempting to speculate that statins may counteract cardiomyocyte injury by inhibition of Rac1-driven pro-oxidative mechanisms. Yet, one should keep in mind that the cardioprotective effect of prototypical antioxidants against anthracycline-induced injury as observed in animal models was inconsistent and, moreover, antioxidants were ineffective in clinical trials.^7^

Regarding their anti-inflammatory properties, statins are described to inhibit nuclear translocation of Nf-kappaB by RhoA/ROCK inhibition, *in vitro*.^130, 131^ RhoA, Cdc42 and Rac1 stimulate the transcriptional activity of Nf-kappaB by favouring phosphorylation of IkappaB alpha.^132^ Accordingly, specific inhibition of these Rho GTPases by clostridial toxins attenuates LPS-stimulated induction of IL6/TNF-alpha as well as Nf-kappaB translocation.^133^ As Nf-kappaB functions as pro-inflammatory transcription factor and is regulated in a Rho-dependent manner, it is feasible that statins interfere with anthracycline-induced cardiac inflammation by inhibition of Nf-kappaB signalling as well as interference with NADPH oxidase. In addition, statins are known to influence TGFbeta/SMAD/CTGF signalling as well as STAT signalling, which both play important roles in fibrosis and cancer development.^134, 135, 136, 137^ Taken together, statins hold the potential to ameliorate anthracycline-induced pro-oxidative, inflammatory and/or fibrotic cardiac responses due to inhibition of Rho-GTPase signalling.

*In vitro* experiments revealed geno- and cytoprotective effects of lovastatin following treatment of non-transformed immortalized and primary cells with doxorubicin or etoposide.^102, 113, 130, 138^ Both drugs have in common that they poison type II topoisomerases, leading to the induction of DSBs. This geno-protective nature of lovastatin points to a link between isoprene precursor depletion (which attenuates Rho GTPase signalling) and the activity of type II topoisomerases. This might be of particular relevance, as catalytic inhibition of TOP2B with dexrazoxane, sobuzoxane (MST-16) and merbarone protects cardiomyocytes from anthracycline-induced cytotoxicity.^41^ In some of these studies, the protective effects of the statin (lowered DSBs levels, attenuated DDR and reduced apoptotic cell death) were mimicked by specific inhibition of Rac1, employing small-molecule inhibitors or clostridial toxins.^102, 113^ The predominant role of Rac1 in the promotion of doxorubicin-induced DSB formation was confirmed in experiments using the Rac1-specific inhibitor EHT1864 and employing a set of immortalized cell lines.^139^ Rac1 inhibition reduced doxorubicin-induced DNA damage formation and response in a cell-type-specific manner, as measured on the basis of S139 phosphorylation of histone H2AX (*γ*H2AX, a well-established surrogate marker of DSBs) and using the comet assay (detection of DNA single-strand breaks and DSBs on single cell level). The geno-protection occurred in a p53-independent manner. This is worth mentioning as the role of p53 in anthracycline-induced cardiomyocyte apoptosis is still contradictory and enigmatic.^26, 140, 141, 142, 143, 144, 145^ Extensive analyses with inhibitors of different Rac1-regulated protein kinases pointed to JNK, ERK, PI3K, PAK and CK1 to be involved in doxorubicin-induced DDR. In addition, Rac1 inhibition reduced phosphorylation of TOP2A on positions S1106 and S1247, pointing to a possible role of Rac1 in the regulation of type II topoisomerase. This finding can be seen in context with the experiments of Lyu *et al.* and Zhang *et al.* who showed cardioprotection from doxorubicin exposure, when type II topoisomerases were inhibited or knocked out *in vitro* and *in vivo*.^54, 58^ The connection between Rac1 and type II topoisomerases is still elusive but underpinned by findings of Sandrock *et al.*^109^ who showed nuclear Rac1 to co-precipitate with type II topoisomerases.

Huelsenbeck *et al.*^113^ demonstrated that a statin co-treatment attenuates acute anthracycline-induced cardiotoxicity in BALB/c mice as mirrored by reduced mRNA levels of pro-fibrotic and pro-inflammatory cytokines. It also protected from doxorubicin-induced sub-acute cardiac damage. In a similar study, atorvastatin protected mice from doxorubicin-induced DNA damage, lipid peroxidation and glutathione depletion.^146^ Using wistar rats, the cardioprotective potential of rosuvastatin (0.5 and 2.0 mg/kg per day; i.p.) was compared to that of carvedilol (1 mg/kg per day; i.p.).^147^ The rats were pre-treated with each drug for 1 month, received a single high dose of doxorubicin (30 mg/kg; i.p.) and were analysed 24 h later. Both drugs similarly prevented doxorubicin-induced increase in blood pressure and caspase-3 protein levels in cardiomyocytes. Like mentioned above, a similar study comparing the protective effects of rosuvastatin and carvedilol 4 weeks after multiple low doses of doxorubicin identified the statin as more potent cardioprotectant than carvedilol.^119^

Yoshida *et al.*^142^ suggested that doxorubicin-induced cardiotoxicity is mediated by oxidative DNA damage followed by ATM/p53-regulated apoptosis. In this study, mice were treated with doxorubicin (6 mg/kg; i.p.) once a week for 4 weeks and co-treated with pitavastatin (3 mg/kg per day). In line with the aforementioned *in vitro* studies of Huelsenbeck *et al.*,^113^ where rat cardiomyocytes (H9c2) were employed, Yoshida *et al.* also found protective effects of a Rac1 inhibition following anthracycline treatment in H9c2 cells. They assigned the protective effect of Rac1 inhibition to its role in NADPH oxidase regulation and oxidative stress and, in line with other data,^148^ suggested that statin-mediated effects are mainly related to inhibition of Rac1 signalling. Accordingly, a cardiac *rac1* knockout protected mice from acute anthracycline-induced cardiotoxicity in a ROS-dependent as well as -independent manner.^149^ Moreover, mice with liver-specific *rac1* knockout are protected from acute anthracycline-triggered DSB formation in hepatocytes too.^150^

Most studies investigating anthracycline effects under situation of inhibition of Rho GTPases point to Rac1-regulated oxidative stress or Rac1-regulated topoisomerase II functions as major factors in acute or sub-acute anthracycline-induced cardiotoxicity. Yet, protection from chronic cardiotoxicity would be clinically more relevant than prevention of acute adverse effects of anthracyclines. Only few animal experiments addressed the possible usefulness of statins in chronic anthracycline-evoked cardiotoxicity so far. In one of those studies, an increase in cardiac pro-inflammatory and pro-fibrotic mRNA levels as well as mitochondrial hyperproliferation was found following anthracycline treatment.^63^ Echocardiographical analyses showed a decrease in the left ventricular posterior wall diameter in doxorubicin-treated mice. These adverse cardiac effects were diminished in animals co-treated with lovastatin. Taken together, statins have the potential to alleviate acute, sub-acute and chronic cardiotoxicity following anthracycline treatment *in vivo*. Due to their pleiotropic effects, statins might counteract the complex dose- and time-dependent mechanisms underlying the cardiac toxicity of anthracyclines. Whereas specific RhoA and/or Rac1 inhibitory drugs are not clinically approved, statins represent Rho inhibitory drugs that are clinically well established, well tolerated and characterized by a wide therapeutic window.

A retrospective clinical cohort study from 2012 found a significant lower risk of heart failure in breast cancer patients receiving statins during the therapy.^151^ Furthermore, recent meta-study demonstrated that statins are at least equally potent as dexrazoxane, beta-blockers or angiotensin antagonists in the prevention of anthracycline-induced cardiotoxicity.^152^ Unfortunately, there are only a few small-scaled prospective studies available that focused on cytoprotective effects of statins in patients receiving anthracycline-based therapies. In one of these studies, 20 patients undergoing anthracycline-based chemotherapy were compared to 20 patients who were co-treated with high doses of atorvastatin (40 mg/kg) during chemotherapy with doxorubicin or idarubicin for up to 6 months.^153^ In the statin co-treated group, no significant change in LVEF was observed, whereas the group that did not receive the statin showed a significant decrease in LVEF (~63% baseline *versus* ~ 55% after therapy). In another study that included 14 patients who received statins during an anthracycline-based chemotherapy and 37 patients who did not,^154^ the statin-treated group showed no significant change in LVEF, whereas those not receiving the statin had a mean LVEF decline of ~7%. Certainly, there is a need of additional randomized prospective studies comprising higher number of patients to validate these cardioprotective effects of statins in anthracycline-based chemotherapy in humans.

## Conclusion and perspective

**Figure 4:**
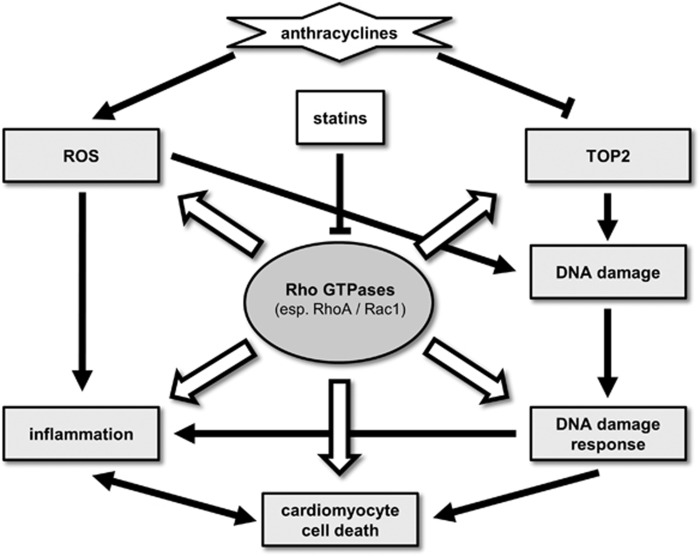
Rho GTPases are involved in the regulation of main factors in anthracycline-induced cardiotoxicity. Anthracyclines induce reactive oxygen species (ROS) by redox cycling as well as Fenton's reaction. ROS induce oxidative DNA damage and are a driver of inflammation. In addition, anthracyclines inhibit type II topoisomerases (TOP2), causing highly cytotoxic DNA double-strand breaks (DSBs), which forcefully trigger a pro-apoptotic DNA damage response and can ultimately result in cardiomyocyte cell death. Rho GTPases such as RhoA or Rac1 are known to play a role in the regulation of cell death and survival. Rac1 is described to regulate ROS production via the NADPH oxidase complex, RhoA and Rac1 participate in the regulation of inflammatory processes by (among others) altering NF-κB signaling after genotoxic insults. Oxidized guanine can act as GEF for nuclear Rac1, making this Rho GTPase a possible novel factor in the regulation of the DNA damage response. In addition, Rac1 is described to interact with type II topoisomerases

Prevention and treatment of anthracycline-induced cardiotoxicity with enalapril, carvedilol and statins are conceivable strategies to reduce the cardiovascular risk. Statins exhibit anti-inflammatory as well as anti-fibrotic properties and interfere with at least two of the suggested main mechanisms involved in anthracycline-caused cardiotoxicity, (i) the generation of ROS and (ii) topoisomerase II inhibition (Figure 4). In animal studies, statins exhibited stronger cardioprotective potential than carvedilol and small-scaled clinical studies showed improvement of cardiac function by statins in anthracycline-treated cancer patients. In addition, statins may sensitize certain tumour entities to chemotherapeutics, which might even further enhance the anti-cancer efficiency of anthracycline-based therapeutic regimen while concomitantly protecting normal tissue, thus widening the therapeutic window. Many of the beneficial effects of a statin treatment could be broken down to the damping of RhoA or Rac1 signalling, making these Rho GTPases preferable targets for future chemo-preventive strategies. Animal studies addressing anthracycline-induced chronic cardiotoxicity in mice lacking Rac1 specifically in cardiomyocytes will clarify the relevance of Rac1 signalling in anthracycline-mediated cardiomyocyte death. Altogether, the pre-clinical and clinical data available so far encourage forthcoming larger phase II studies that address the clinical efficacy of statins for normal tissue protection in anthracycline-based therapeutic regimens.
